# The characterization and structural basis of a human broadly binding antibody to HBV core protein

**DOI:** 10.1128/jvi.01694-24

**Published:** 2024-11-27

**Authors:** Hu Yan, Congcong Liu, Yuxiao Li, Shilong Tang, Huimin Guo, Bing Zhou, Qing Fan, Haiyan Wang, Xiangyang Ge, Xin Wang, Xuejiao Liao, Jin Li, Zheng Zhang, Bin Ju

**Affiliations:** 1 Institute for Hepatology, National Clinical Research Center for Infectious Disease, Shenzhen Third People’s Hospital 255310 , Shenzhen, Guangdong Province, China; 2 The Second Affiliated Hospital, School of Medicine, Southern University of Science and Technology 255310 , Shenzhen, Guangdong Province, China; 3 Guangdong Key Laboratory for Anti-infection Drug Quality Evaluation, Shenzhen, Guangdong Province, China; 4 Shenzhen Research Center for Communicable Disease Diagnosis and Treatment, Chinese Academy of Medical Sciences 623677 , Shenzhen, Guangdong Province, China; Lerner Research Institute, Cleveland Clinic, Cleveland, Ohio, USA

**Keywords:** hepatitis B virus, core protein, human monoclonal antibody, structure analysis

## Abstract

Hepatitis B virus (HBV) core protein (HBc) plays a crucial role in the virus life cycle, making it an important detection marker for HBV infection and a potential target for treatment. However, several commercially available monoclonal antibodies (mAbs) or polyclonal antibodies (pAbs) targeting HBc have certain limitations in detecting HBV across different genotypes in various biochemical assays, such as enzyme-linked immunosorbent assay, western blot, immunofluorescence assay, flow cytometry, and immune spot assay. In this study, we identified 12 human anti-HBc mAbs and evaluated their potential application in multiple biochemical assays. These mAbs mainly recognized the epitopes near residues 20–22 amino acids (a.a.) and 77–78 a.a. of HBc, demonstrating a broadly cross-genotypic activity. Notably, three Group II mAbs, named cAbA1, cAbD4, and cAbF9, displayed excellent capacities for the detection of HBV infection in all tested biochemical assays. Furthermore, we determined a 3.22 Å of cryo-electron microscopy (cryo-EM) structure of the fragment of antigen binding (Fab) of cAbD4 complexed with HBc dimer, which was the highest resolution of the structural model for Fab-HBc to date. Collectively, our findings provided excellent and reliable antibody candidates for live HBV detection and revealed the recognition mechanism and the detailed interaction information of a potent human anti-HBc mAb binding to the spike tips of HBc dimer.

**IMPORTANCE:**

The lack of excellent detection Abs for live hepatitis B virus (HBV) infection and high-resolution structures of the Ab-HBV core protein (HBc) complex largely limited the development of HBV-related research. This study reports a panel of anti-HBc monoclonal antibodies (mAbs) with excellent capacities for detecting HBV infection in multiple biochemical assays and determines a 3.22 Å of cryo-EM structure of HBc with a potent binding mAb. These findings provide excellent and reliable detecting tools for HBV-related research and promote the understanding of the recognition mechanism of anti-HBc mAbs to HBc particles.

## INTRODUCTION

Hepatitis B virus (HBV) infection remains a major public health threat, with approximately 254 million people living with chronic HBV infection, which results in 1.1 million deaths mainly due to HBV-associated cirrhosis and hepatocellular carcinoma in 2022 (1). Currently, despite the available treatment regimens such as nucleos(t)ide analogs (NAs) and interferon-α (IFN-α), which can effectively suppress HBV DNA replication and disease progression, achieving a functional cure for chronic HBV infection remains rare (2). There is still an urgent need to develop more strategies for detecting and combating the HBV infection.

HBV, a partially double-stranded DNA virus, possesses a spherical structure with a diameter of approximately 42 nm. Its inner capsid is assembled by core protein (HBc), which is composed of 183 amino acids (a.a.) (HBc_1-183_) among most HBV genotypes. The HBc contains an N-terminal assembly domain for capsid forming (1–149 a.a.) and an arginine-rich C-terminal domain for nucleic binding and nuclear localization (150–183 a.a.) (3). The N-terminal domain has five α helices and forms dimer with four-helix bundles at the interface projecting as spikes through hydrophobic interaction (4). Typically, 120 dimers with T = 4 icosahedral symmetry could self-assemble into capsids (5). These capsids that encapsidate viral RNA and polymerase, not only provide a contained compartment for reverse transcription to DNA but also facilitate nucleocapsid envelopment and virions formation (6–9). Moreover, HBc also regulates capsid transport into the nucleus and is associated with cccDNA formation during infection (10). In addition to its structural role and regulatory function in most stages of the viral life cycle, HBc potentially plays a role in the pathogenesis of HBV-associated disease by suppressing the host immune response or activating several signal pathways such as MAPK pathways and Wnt/β-catenin pathways (11–13). Collectively, the multifunctionality of HBc raises that it could be a crucial target for HBV detection and intervention.

Nowadays, numerous monoclonal antibodies (mAbs) against HBc have been identified (14–16). Some mAbs can be utilized in part of biochemical assays such as western blot, immunofluorescence assay (IFA), flow cytometry, and immune spot for HBV detection. However, the commercial mAbs with widespread applicability in detecting HBV infection across different genotypes are rare, which underscores the need for the development of effective detection mAbs for HBV-related research. In general, the excellent capacity of mAbs for HBV detection is closely correlated with their binding epitopes. Therefore, identifying the epitopes recognized by some excellent mAbs is of great guidance for the development of HBV detection antibodies against HBc.

Cryo-electron microscopy (cryo-EM) analysis has revealed seven epitopes of capsid recognized by anti-HBc mAbs (17, 18). Six are on the capsid spike tips known as the ‘‘immunodominant loop’’ (~residues 78–83 a.a.), and another epitope is on the floor around the symmetry axes. Although the six epitopes (residues 78–83 a.a.) on the spike are close together and nearly overlapping, they interact with their respective fragment of antigen binding (Fab) of mAbs in different binding aspects with diverse affinities (19). Similarly, in another research of polyclonal antibodies (pAbs), different epitopes were identified by cryo-EM analysis of human Fabs-labeled capsids (18). However, only lower-resolution structures of mAbs complexed with HBc have been reported until now (20, 21), which also largely limited the development of HBV-related research.

In this study, we identified 12 human anti-HBc mAbs and investigated their binding epitopes and potential applications in multiple biochemical assays. Most of the mAbs exhibited a broadly cross-genotypic activity and significantly recognized the overexpression of HBc in flow cytometry and IFA. Moreover, some of the mAbs could be used in the western blot analysis, suggesting their binding capacity to linear epitopes. Notably, 3 mAbs (cAbA1, cAbD4, and cAbF9) could detect the HBV infection of live virus in all above-mentioned assays as well as immune spot assays. Competition ELISA, truncation detection, and alanine-scanning experiment were performed to classify these 12 mAbs into 3 groups according to their predicted binding epitopes. Furthermore, the high-resolution cryo-EM structure of HBc dimer labeled with the Fab of cAbD4 was determined, revealing the detailed interaction information and key residues at their interface. Overall, we provided a series of human anti-HBc mAbs with a broadly cross-genotypic activity, which was of significance for the HBV detection in multiple biochemical assays and other potential HBV-related research.

## RESULTS

### The identification of 12 human anti-HBc mAbs

**Fig 1:**
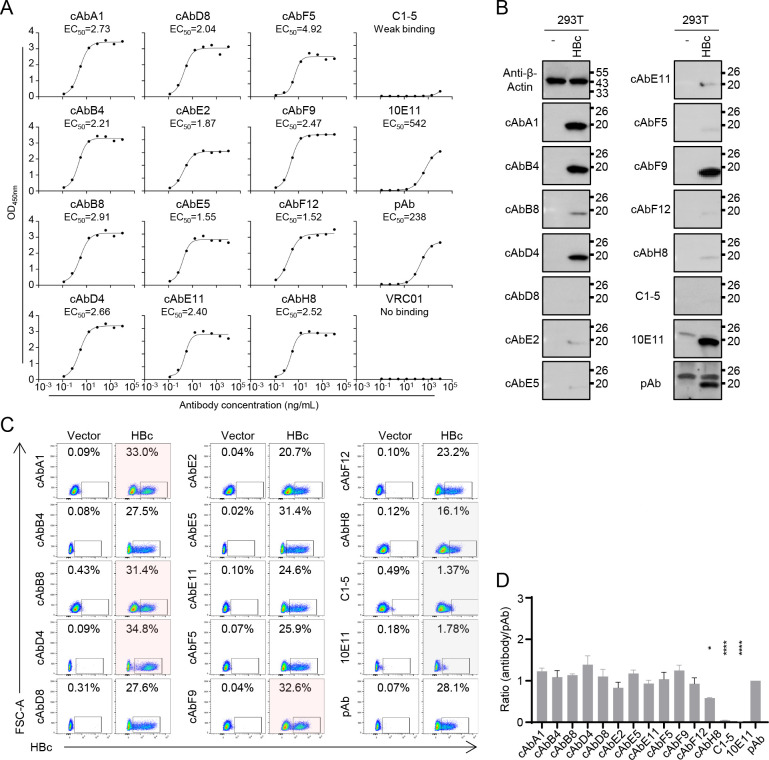
The identification and detection of 12 human mAbs binding to HBc from genotype C in multiple biochemical assays. (**A**) ELISA analysis of the binding activities of 12 human mAbs, C1-5, 10E11, and pAb to HBc_1-143_ protein from genotype C. The full-length HBc protein from genotype C overexpressed in 293T cells was analyzed by western blot (**B**) and flow cytometry (**C**). (**D**) The ratio of HBc-positive population recognized by mAbs compared to pAb. C1-5, 10E11, and pAb are commercial antibodies. VRC01 is an HIV-1 mAb, as a negative control. Data are presented as mean ± SD. *P < 0.05; ****P < 0.0001. Statistical analysis was determined by one-way ANOVA. Each experiment was independently performed at least twice and one representative result was shown.

We isolated numerous human mAbs against HBc from two chronic HBV patients with HBV genotype B infection, among them 12 mAbs (cAbA1, cAbB4, cAbB8, cAbD4, cAbD8, cAbE2, cAbE5, cAbE11, cAbF5, cAbF9, cAbF12, and cAbH8) exhibited strongly binding activities to the HBc_1-143_ of genotype C HBV in the enzyme-linked immunosorbent assay (ELISA) (Fig. 1A). By contrast, VRC01, a HIV-1 mAb as the negative control in this study, showed no binding activity to HBc. Two commercial mouse mAbs (C1-5 and 10E11) and a rabbit pAb served as positive controls. As shown in Fig. 1A, C1-5 weakly bound to HBc_1-143_ of genotype C HBV. All 12 human mAbs identified in this study demonstrated a nanogram level of 50% of maximal effect concentration (EC_50_) ranging from 1.52 to 4.92 ng/mL, which were much lower than that of 10E11 (542 ng/mL) and the commercial pAb (238 ng/mL). These results indicated that these 12 human mAbs possessed a strong binding activity to HBc.

**Fig 2:**
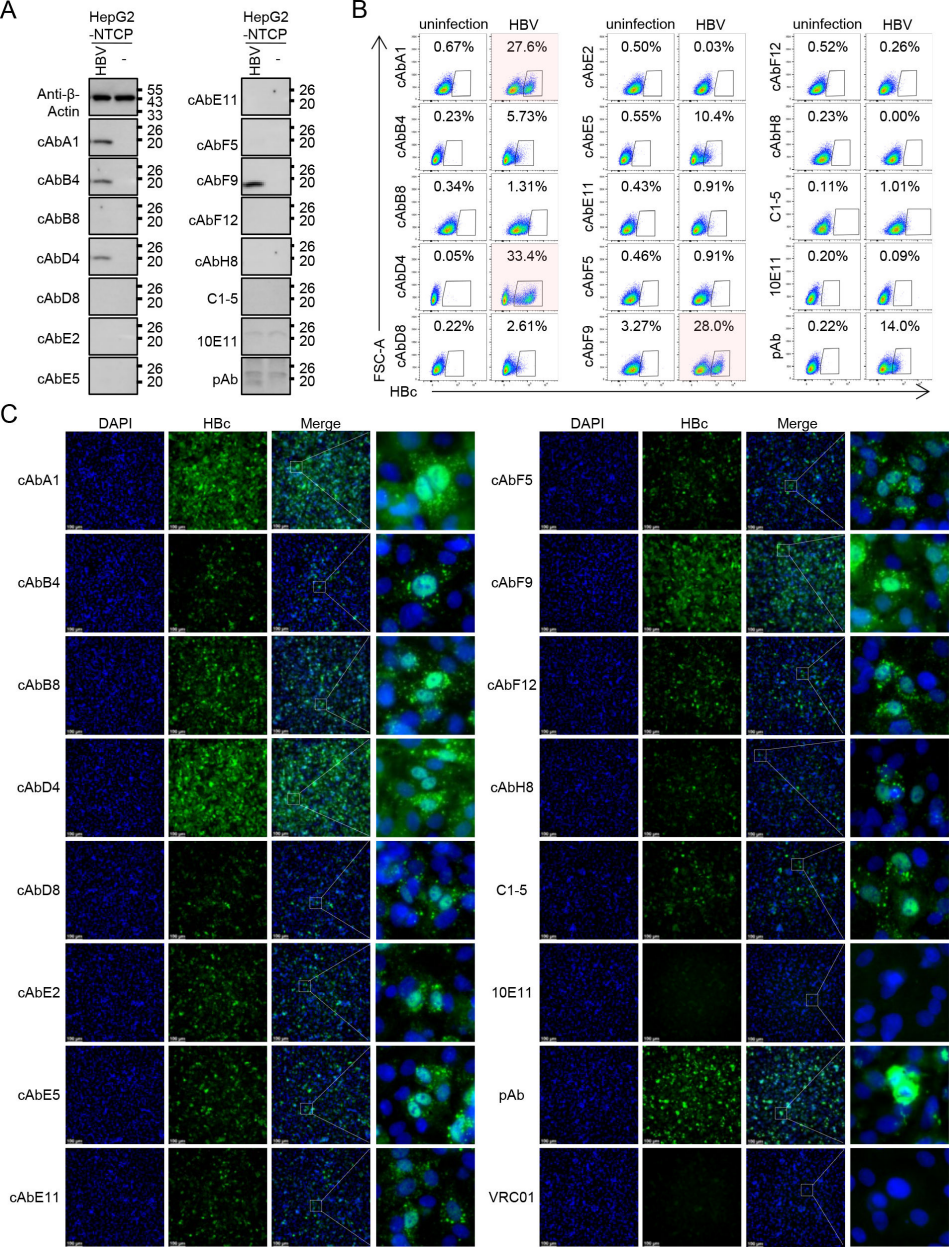
The application of 12 human anti-HBc mAbs in detecting the live HBV infection. The HBc expression in HepG2-NTCP cells infected with live HBV from genotype D after 5–7 days was detected using 12 human mAbs, C1-5, 10E11, and pAb by western blot (**A**), flow cytometry (**B**), and IFA (**C**). Scale bar: 100 µm. Each experiment was independently performed at least twice and one representative result was shown.

**Fig 3:**
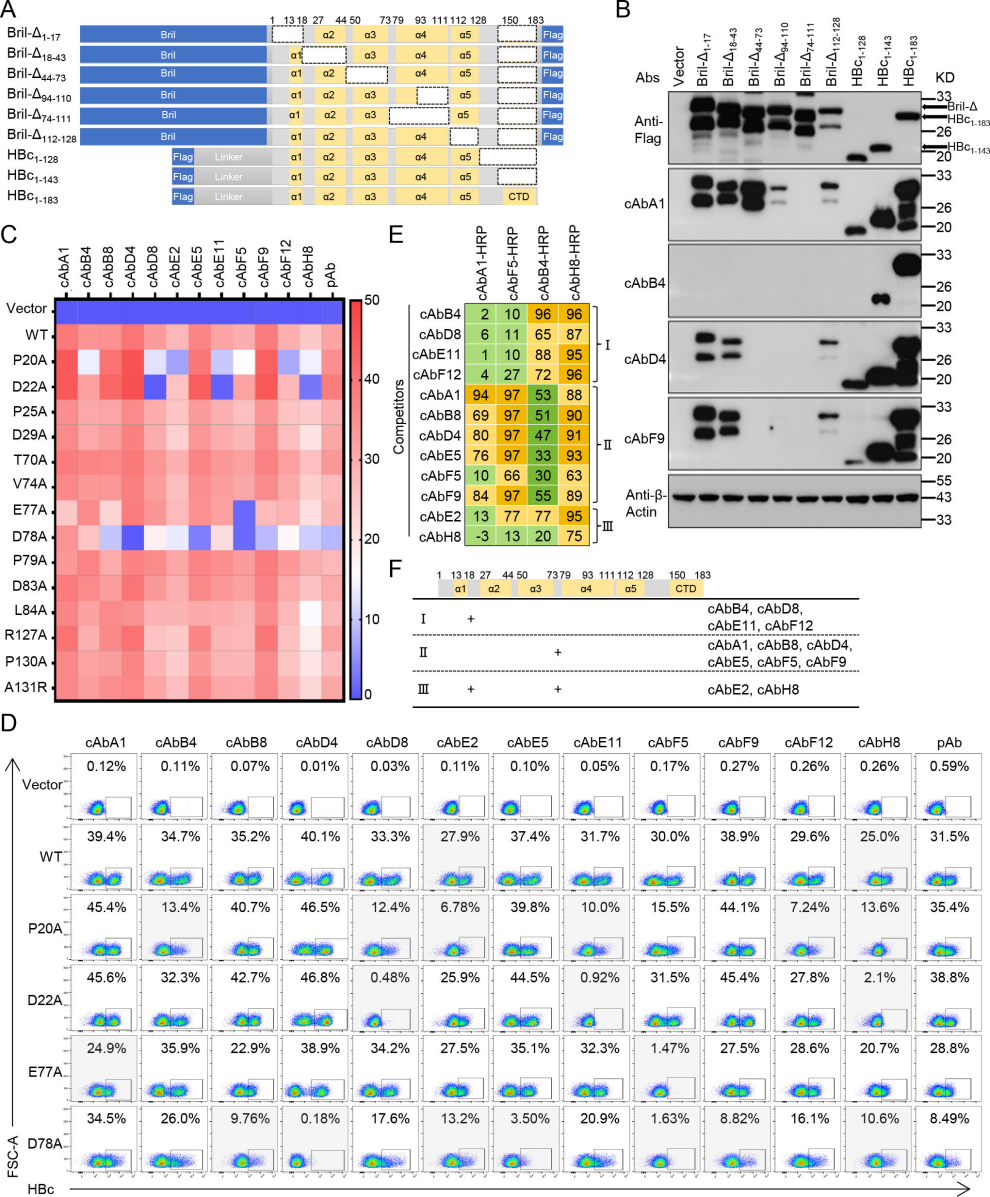
The binding epitope characteristics of 12 human anti-HBc mAbs. (**A and B**) Flag tag labeled different HBc truncations fused with or without Bril were overexpressed in 293T cells and detected by western blot using anti-Flag, cAbA1, cAbB4, cAbD4, and cAbF9. (**A**) Schematic of a Flag-tag HBc_1-183_ and 8 truncations. (**B**) Western blot analysis of the binding activities of antibodies to different HBc truncations. (**C, D**) The residues 20, 22, 25, 29, 70, 74, 77, 78, 79, 83, 84, 127, and 130 a.a. of HBc from genotype D mutated to alanine and residue 131 a.a. were mutated to arginine, overexpressed in 293T cells, and detected by flow cytometry using 12 human mAbs and pAb. (**C**) The percent of full-length (wild type, WT) and mutated HBc detected by antibodies were displayed by heat map. (**D**) Representative FACS plots to identify the proportion of the HBc-specific population. (**E**) Results of competition ELISA. cAbA1, cAbF5, cAbB4, and cAbH8 coupled with HRP were used for binding to HBc_1-143_. Light green, inhibition <30% (no competition); green, 31%–60% (weak); light orange, 61%–90% (moderate); and orange, ≥91% (strong). The data are means of two independent experiments. (**F**) Schematic of potential recognition epitopes of 12 human anti-HBc mAbs. The experiments in B, C, and D were independently performed at least twice and one representative result was shown.

**Fig 4:**
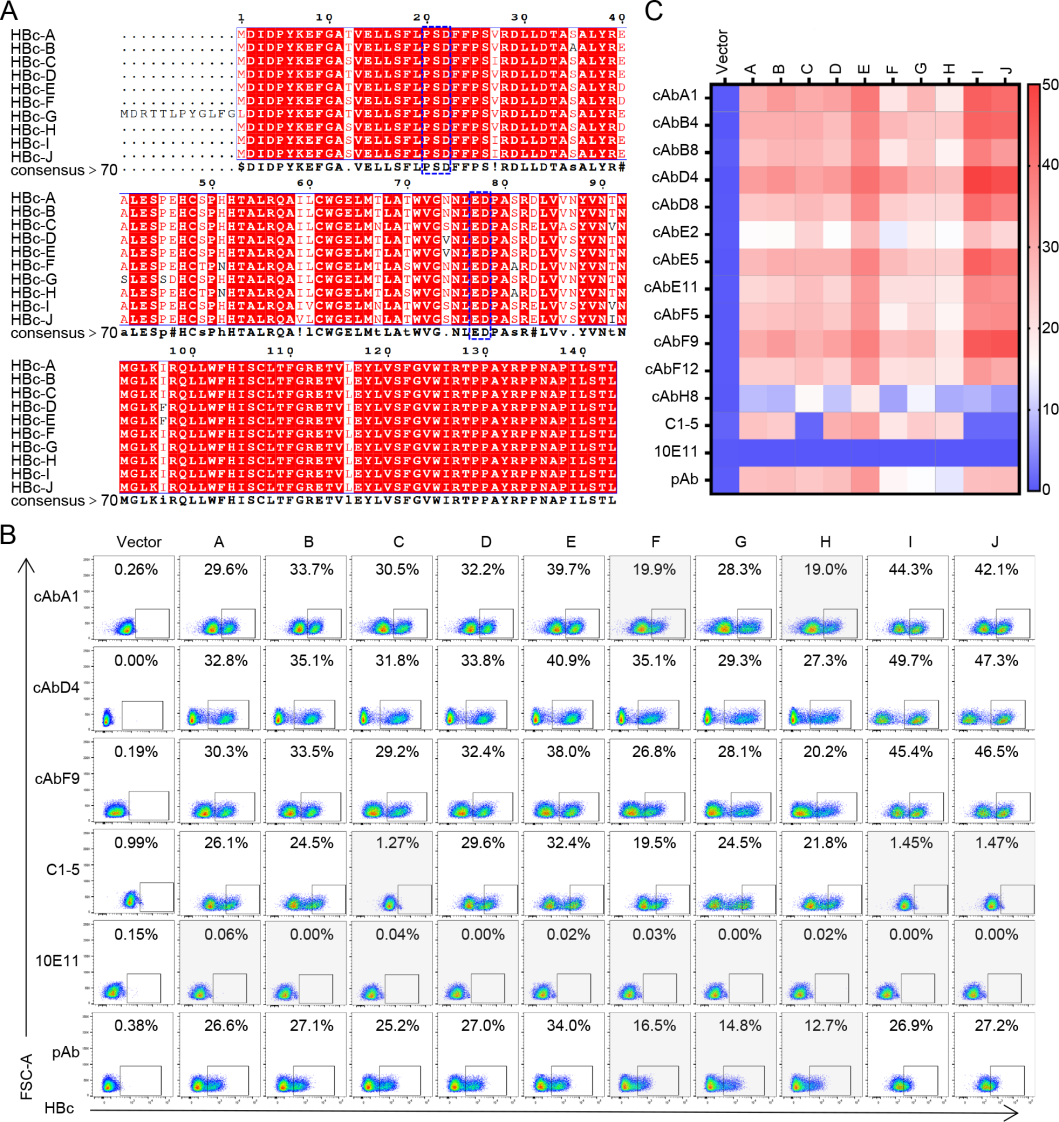
Cross-reactivity of 12 human mAbs to different HBc from genotypes A–J. (**A**) The amino acid alignment of HBc_1-143_ from genotypes A–J HBV was performed by Clustal W and created by ESPript 3.0. (**B**) The reactivity of antibodies to detect different genotypes of HBc was evaluated by flow cytometry. (**C**) The percentage of HBc-specific cells detected in (**B**) was shown by heat map. Experiments were independently performed at least twice and one representative result was shown.

To further explore the potential applications of these 12 human mAbs, the western blot analysis was performed with overexpressed HBc of genotype C in 293T cells (Fig. 1B). cAbA1, cAbB4, cAbD4, cAbF9, 10E11, and pAb strongly detected the main band of HBc, while cAbB8 weakly recognized it, suggesting these antibodies could identify linear epitopes. However, the detection of 10E11 and pAb showed some non-specific bands, especially pAb. Moreover, all 12 human mAbs were able to detect the HBc expressed in 293T cells by flow cytometry (Fig. 1C). Compared with pAb, 12 human mAbs except cAbH8 exhibited a comparable percentage of HBc-specific cells (Fig. 1C and D). Meanwhile, an obviously positive population was classified using cAbA1, cAbB8, cAbD4, and cAbF9. By contrast, no positive population was found using mouse mAbs (C1-5 and 10E11). Similarly, all 12 human mAbs and pAb significantly detected the expressed HBc of genotype C in cells by IFA (Fig. S1A). However, the detection of pAb also showed a significant non-specific background (Fig. S1B). Neither C1-5 nor 10E11 recognized the expressed HBc in 293T cells. Compared with the positive signal of HBc detection by C1-5 in other studies (10, 22), our results showed that it could not detect the HBc expression of genotype C, which might be due to the use of different HBV genotypes. Overall, we identified 12 human anti-HBc mAbs, among which cAbA1, cAbD4, and cAbF9 could be well applied in multiple biochemical assays in the overexpression system.

### The detection of HBV infection by 12 human anti-HBc mAbs in multiple biochemical assays

To further investigate the potential application of these 12 human anti-HBc mAbs in detecting live virus infection, HepG2-NTCP cells were infected by genotype D HBV purified from Hep AD38 cells and then tested in a series of biochemical assays. Similar to the overexpression experiments, cAbA1, cAbB4, cAbD4, and cAbF9 were able to detect the main band of HBc in western blot assay after HBV infection (Fig. 2A). C1-5, 10E11, and pAb failed to recognize HBc except for some non-specific bands, potentially attributed to the low expression of HBc or different HBV genotypes. Moreover, the use of cAbA1, cAbD4, and cAbF9 revealed the distinct populations of HBV-infected cells, indicating their capability to detect the HBc in flow cytometry analysis (Fig. 2B). Recent studies indicated that HBc was in flux between cytoplasmic and nuclear in a time- and concentration-dependent manner (23). Subsequent IFA results revealed that all antibodies except for 10E11 recognized both cytoplasmic and nuclear HBc (Fig. 2C). Notably, cAbA1, cAbD4, and cAbF9 exhibited more intensive fluorescence signals compared to other antibodies. These results demonstrated that cAbA1, cAbD4, and cAbF9 were suitable for HBc detection of HBV-infected cells in western blot, flow cytometry, and IFA.

As HBV infection does not result in plaque formation, detecting the infected cells by focus reduction test poses challenges. Currently, the detection of HBV infection is indirectly determined by the assessment of HBsAg or HBeAg secretion *in vitro* assay (24–26). To detect the HBV-infected cells, we performed an immune spot assay and analyzed by Cytation7 (Fig. S2). cAbA1, cAbD4, and cAbF9 could clearly identify the single HBV-infected cell in a viral dose-dependent effect, while cAbB8 and cAbE5 only recognized the infected cells under high HBV titers, indicating the different sensitivities of these human mAbs. By contrast, C1-5 and pAb failed to show a viral dose-dependent pattern and exhibited significant non-specific background signals. These results highlighted that cAbA1, cAbD4, and cAbF9 could effectively detect the HBV-infected cells in immune spot analysis.

### The binding epitope characteristics of human anti-HBc mAbs

To identify the binding epitopes of these human anti-HBc mAbs, we generated eight HBc truncations (4). Bril-Δ_1-17_ (deletion 1–17 a.a. of HBc_1-183_), Bril-Δ_18-43_ (deletion 18–43 a.a. of HBc_1-183_), Bril-Δ_44-73_ (deletion 44–73 a.a. of HBc_1-183_), Bril-Δ_94-110_ (deletion 94–110 a.a. of HBc_1-183_), Bril-Δ_74-111_ (deletion 74–111 a.a. of HBc_1-183_), and Bril-Δ_112-128_ (deletion 112–128 a.a. of HBc_1-183_) were fused with N terminal Bril to enhance the stability (27) and with C terminal Flag to be detected, and HBc_1-128_, HBc_1-143_, and the full-length HBc_1-183_ were fused with N terminal Flag and a long linker (Fig. 3A). Then these diverse truncations of HBc were expressed in 293T cells, respectively, and detected by several mAbs in western blot assay. All the recombinant HBc could be recognized by anti-flag, indicating that they were all successfully expressed (Fig. 3B). Based on the result above (Fig. 1B), cAbA1, cAbB4, cAbD4, and cAbF9 were performed to identify the different truncations of HBc in immunoblot assay (Fig. 3B). cAbA1 could detect most of HBc truncations except Bril-Δ_74-111_, which had a deletion of residues 74–93 a.a. compared with Bril-Δ_94-110_, indicating that its binding epitopes were around this region. cAbB4 only recognized the HBc_1-143_ and HBc_1-183_. cAbD4 and cAbF9 could not bind to the Bril-Δ_44-73_, Bril-Δ_94-110_, and Bril-Δ_74-111_, indicating their epitopes were among residues 44–111 a.a. of HBc.

To map these epitopes more precisely, we conducted alanine-scanning experiments using a series of HBc variants with substitutions covering the reported epitopes (18, 28) (Fig. 3C and D; Fig. S3). In line with the detection of HBV infection above, all 12 human mAbs and pAb could recognize the positive population of wild-type (WT) HBc of genotype D, with cAbE2 and cAbH8 showing a slightly lower percentage of HBc-specific cells. Among these mutated HBc, the positive population of P20A, D22A, E77A, and D78A was not distinctly classified by some mAbs and pAb in flow cytometry. cAbB4, cAbD8, cAbE11, and cAbF12 mainly bound near residues 20–22 a.a. of HBc. cAbB8, cAbD4, cAbE5, cAbF5, and cAbF9 are mainly recognized the position near residues 77–78 a.a. of HBc. cAbE2 could target both residues of HBc. In consistent with the epitopes detected in Fig. 3B, cAbA1 could recognize all listed mutated HBc but did not obviously separate the positive of E77A with a slightly lower proportion compared with the others, suggesting its epitope might be near the residue E77 of HBc (Fig. 3C and D). Moreover, cAbH8 could weakly detect the positives of all mutated HBc, especially if it barely recognized the positives of P20A, D22A, and D78A, indicating that these positions might be recognized by cAbH8. These results suggested that residues 20–22 a.a. and 77–78 a.a. of HBc might be the immunodominant epitopes significantly recognized by these human mAbs.

It is generally believed that a single mutation has little effect on capsid assembly; however, a certain site mutation can still affect its spatial conformation in some cases. For example, a D78S mutation of HBc leads to a more open conformation in the spike tip of HBc dimer compared with WT (29), which might influence some mAbs targeting sites far away from the residue D78. To redetect the binding epitopes of these human mAbs, we further performed competition ELISA assays, in which HBc_1-143_ was pre-incubated and followed by a mixture of given HRP-labeled mAbs and an unlabeled antibody (Fig. 3E). Based on the results of mAbs recognition to the different truncated and alanine-mutated HBc variants, cAbA1 and cAbF5 recognizing near residue E77, cAbB4 targeting residue P20, and cAbH8 probably binding to both P20 and E77 were labeled with HRP and considered as the reference mAbs in competition ELISA. As expected, all four tested mAbs (cAbA1, cAbF5, cAbB4, and cAbH8) well blocked the binding of their autologous HRP-labeled mAbs. Thereafter, 12 human anti-HBc mAbs were classified based on their obvious competition rate of over 60%. cAbB4, cAbD8, cAbE11, and cAbF12 moderately or strongly competed with cAbB4 and cAbH8 yet not with cAbA1 and cAbF5. Differently, cAbA1, cAbB8, cAbD4, cAbE5, cAbF5, and cAbF9 moderately or strongly competed with cAbA1, cAbF5, and cAbH8, yet not with cAbB4. By contrast, cAbE2 and cAbH8 competed with cAbF5, cAbB4, and cAbH8. Taken together, based on the recognition patterns in HBc truncation detection, alanine-scanning experiment, and competition ELISA, these 12 human mAbs could be classified into three groups: Group I (cAbB4, cAbD8, cAbE11, and cAbF12), Group II (cAbA1, cAbB8, cAbD4, cAbE5, cAbF5, and cAbF9), and Group III (cAbE2 and cAbH8) (Fig. 3F).

### Human anti-HBc mAbs with a broadly cross-genotypic reactivity

Based on the divergence of >7.5% across the entire genome, HBV has been classified into nine major genotypes (A–I) and one putative genotype (J) (30). HBc_1-143_ recognized by 12 human mAbs was highly conserved among different genotypes with 89.03% of identity (Fig. 4A). In particular, the residues 20–22 a.a. and 77–78 a.a. of HBc were identical among tested A-J genotypes, suggesting that these 12 human mAbs might bind to all genotypes of HBc. To further assess the cross-reactivity, HBc from genotypes A–J were individually expressed in 239T cells and then measured using 12 human mAbs, C1-5, 10E11, and pAb in flow cytometry analysis (Fig. 4B and C; Fig. S4). In consistent with the result of sequence alignment analysis on the residues 20–22 a.a. and 77–78 a.a. of HBc, most human mAbs except cAbE2 and cAbH8 were able to detect all different genotypes of HBV. cAbA1, cAbB8, cAbE2, cAbF5, and cAbH8 recognized a slightly low proportion or could not distinctly separate the positive population of HBc-specific cells from genotype F or H, indicating that in addition to the residues 20–22 a.a. and 77–78 a.a., other residues of HBc also might affect the binding of these human mAbs. Of note, cAbD4 showed superior activity in distinguishing the positive population of HBc of genotypes A–J compared to the other mAbs. By contrast, the commercial mouse mAb C1-5 could not recognize the HBc of genotypes C, I, and J, and the pAb weakly detected the HBc of genotypes F–H. Similar to the aforementioned test (Fig. 1C), 10E11 either could not recognize the HBc or was not suitable for the flow cytometry analysis. These findings demonstrated that most of the human anti-HBc mAbs identified in this study exhibited a broadly cross-genotypic activity.

Taken together, most human mAbs, particularly cAbD4 with a broadly cross-genotypic activity, were able to recognize the HBc overexpression and HBV infection in multiple biochemical assays, such as ELISA, western blot, IFA, flow cytometry, and immune spot assay. The overall application and characteristics of these 12 human anti-HBc mAbs are summarized and detailed in Table 1.

**TABLE 1:** The potential applications of human anti-HBc mAbs in multiple biochemical assays^*h*^

	HBc overexpression					HBV infection			
	ELISA^*a*^	Western blot^*b*^	FCM^*c*^	IFA^*d*^	Cross-reactivity^*e*^	Western blot^*b*^	FCM^*f*^	IFA^*d*^	FFU^*g*^
cAbA1	+++	+++	+++	+++	++	+++	++	+++	+++
cAbB4	+++	+++	++	+++	++	++	-	+	-
cAbB8	+++	+	+++	++	++	-	-	++	+
cAbD4	+++	+++	+++	+++	+++	++	+++	+++	+++
cAbD8	+++	-	++	++	++	-	-	+	-
cAbE2	+++	-	++	++	+	-	-	+	-
cAbE5	+++	-	++	++	++	-	-	+	+
cAbE11	+++	-	++	++	++	-	-	+	-
cAbF5	+++	-	++	++	++	-	-	+	-
cAbF9	+++	+++	+++	+++	++	+++	++	+++	+++
cAbF12	+++	-	++	++	++	-	-	+	-
cAbH8	+++	-	+	++	+	-	-	+	-

^*a*^ +++, the OD_450nm_ value of 10 µg/mL mAbs binding to HBc_1-143_＞ 3.

^*b*^ +++, strong band; ++, moderate band; +, weak band; -, no band.

^*c*^ +++, detected an obviously positive group of HBc with a similar percent to pAb; ++, detected a moderately positive group of HBc with a similar percent to pAb; +, detected a moderately positive group of HBc with a lower percent compared to pAb.

^*d*^ +++, strong fluorescence intensity; ++, moderate fluorescence intensity; +, weak fluorescence intensity.

^*e*^ +++, detected all the listed HBV genotypes with an obviously positive group of HBc; ++, detected all the listed HBV genotypes with a moderate positive group of HBc; +, detected less than 50% of all the listed HBV genotypes with an obviously positive group of HBc.

^*f*^ +++, detected an obviously positive group of HBc with strong fluorescence intensity; ++, detected an obviously positive group of HBc with moderate fluorescence intensity; -, undetected an obviously positive group of HBc.

^*g*^ +++, detected the single HBV-infected cells in a virus-dose-dependent effect; ++, just detected the single HBV-infected cells during a high-dose virus infection; -, undetected the single HBV-infected cells.

^*h*^ FCM, flow cytometry; FFU, focus forming units.

### The structure of HBV capsid-cAbD4 Fab complex

**Fig 5:**
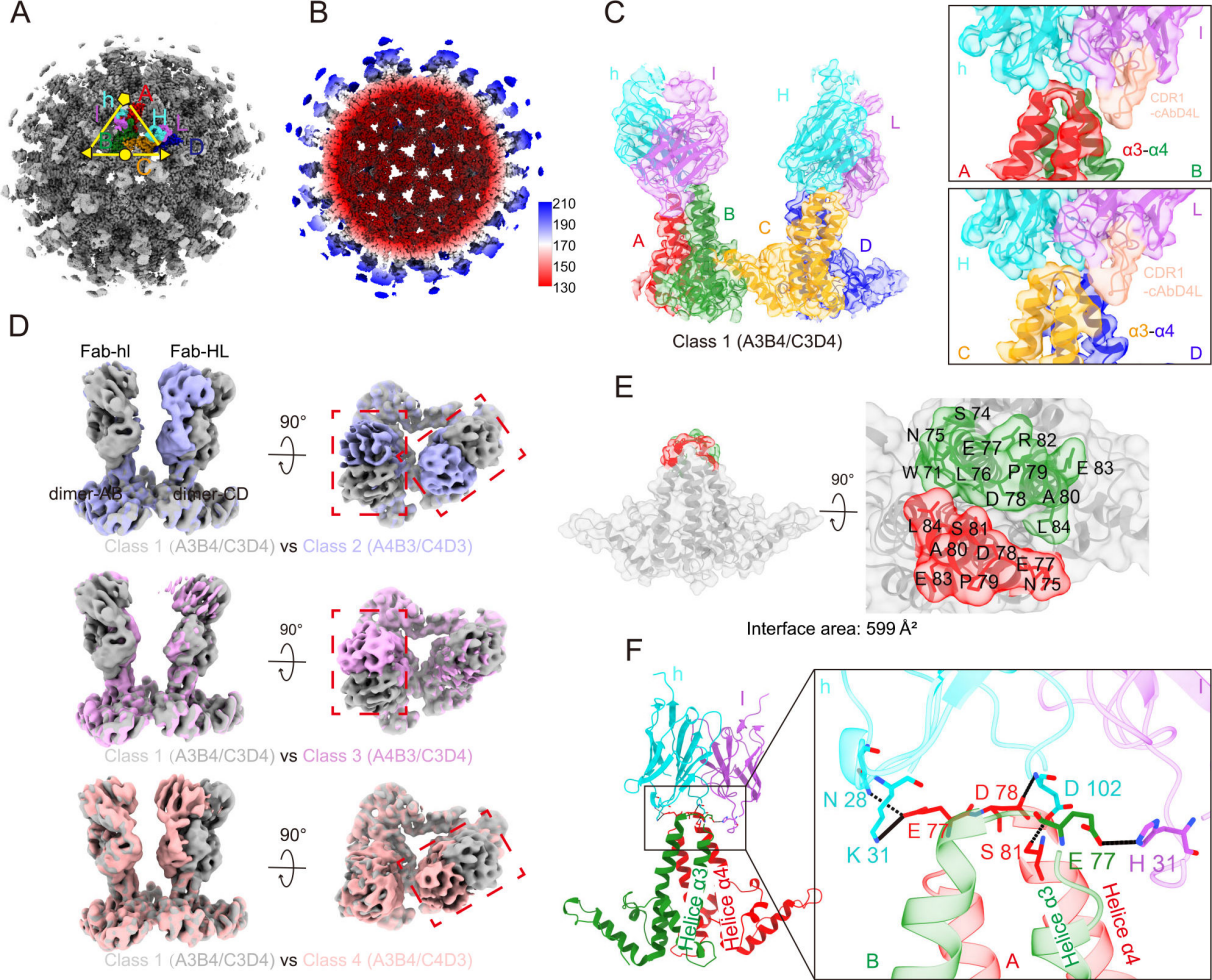
Cryo-EM structure of the HBV capsid-cAbD4 Fabs complex and the ASUs. (**A**) The density map of the HBV capsid-cAbD4 Fabs complex. The fivefold, threefold, and twofold axes are indicated by a pentagon, triangle, and circle, respectively. (**B**) The external surface of the HBV capsid-cAbD4 Fabs complex is displayed. (**C and D**) Results of the 3D classification of the ASUs. (**C**) The ASU adopts model A3B4/C3D4, with CDR1-cAbD4L positioned on the side of α3 helix of chain A and the α4 helix of chain B. (**D**) Comparison of the ASUs adopting models A4B3/C4D3, A4B3/C3D4, and A3B4/C4D3 with the ASU adopting model A3B4/C3D4. Fabs located on the two different sides of the dimer tips are highlighted by red boxes. (**E**) The footprint of cAbD4 Fabs on the dimer tip. The footprint of chain A is represented by red, and the footprint of chain B is represented by forest green. The relevant amino acids are marked accordingly. (**F**) The amino acids involved in the formation of hydrogen bonds (indicated by dashed lines) and salt bridges (indicated by solid lines) within the interaction region between dimer-AB and Fab-hl are determined using PDBePISA calculations (https://www.ebi.ac.uk/pdbe/pisa/). All of the ASUs are displayed in color, with chains A, B, C, and D colored in red, forest green, orange, and blue, respectively. The heavy chains and light chains are colored in cyan and magenta, respectively.

To clarify the structural basis of the interaction between cAbD4 mAb and HBc, the structure of the HBV capsid-cAbD4 Fab complex was determined by cryo-EM. The whole structure of the capsid-cAbD4 Fab complex was determined at 3.44 Å of resolution (Fig. 5A; Fig. S5; Table S1). As previously revealed (31), the capsid-cAbD4 Fab complex exhibits an icosahedral symmetry (T = 4) with a radius of approximately 180 Å (Fig. 5B). Each asymmetric unit (ASU) comprises two dimers of HBc molecules, which contain five helices (α1-α5) and two cAbD4 Fabs, with each cAbD4 Fab binding to the tip of one dimer. Two HBc molecules in the pentamers are denoted as A and B, whereas another two HBc molecules in the adjacent trimer are denoted as C and D. The heavy and light chains of cAbD4 Fab binding with dimer-AB are denoted as h and l, while those binding with dimer-CD are denoted as H and L (Fig. 5A). To improve the qualities of the densities for cAbD4 Fabs, sub-particles were extracted with an ASU mask. After the 3D classification of these sub-particles, Class 1 map represents a complete ASU, in which two complementarity determining region (CDR) 1 loop (residues 27–37 a.a.) of cAbD4 Fab light chain (CDR1-cAbD4L) are on the A-α3/B-α4 side of dimer-AB and C-α3/D-α4 side of dimer-CD (model as A3B4/C3D4), respectively (Fig. 5C). In addition, Class 2, Class 3, and Class 4 maps represent the different modes of ASUs, adopting as A4B3/C4D3, A4B3/C3D4, and A3B4/C4D3, respectively (Fig. 5D).

To further investigate the epitopes of cAbD4 Fabs on HBc dimers, local refinement of these four maps was performed using property masks, resulting in maps at a resolution of 3.68, 3.22, 3.61, and 3.51 Å, respectively (Fig. S5B and S6). The structure for the ASU exhibits a similar conformation compared to the published cryo-EM (PDB: 3J2V) or crystal structure (PDB: 1QGT) of HBV capsid, with a root-mean-square deviation (RMSD) of 1.983 over 553 atoms or 1.784 over 552 atoms, respectively (Fig. S7). Subsequently, a more detailed analysis of interactions between cAbD4 Fab-hl and HBc dimer-AB, adopted as the A4B3 model, was further conducted. cAbD4 Fab was found to have a binding footprint similar to that of 3105 Fab, which bound to the tips of dimers containing residues 77–80 and 83–84 (28), defined as human epitope 4 (he4) (18), with an interface area of 599 Å^2^ (Fig. 5E; Fig. S8). cAbD4 Fab utilizes two residues on heavy chain, namely N28 and D102, to form three potential hydrogen bonds with three residues on chain A (E77, D78, and S81) of HBc. In addition, the negatively charged residues E77 in both chains A and B formed salt bridges with the positively charged residues K31 in the heavy chain and H31 in the light chain (Fig. 5F; Fig. S9).

Overall, the structural analysis of HBV capsid-cAbD4 Fab complex demonstrated various types of binding modes in a dimer unit of Fab with HBc, such as Class 1 (A3B4/C3D4), Class 2 (A4B3/C4D3), Class 3 (A4B3/C3D4), and Class 4 (A3B4/C4D3), and also revealed the exact recognition epitopes of cAbD4, which was the most potent and broad binding mAb to HBc identified in this study.

## DISCUSSION

The related detections of the expression and nuclear location of HBc were previously performed using some commercial pAbs with significant limitations. For example, the Dako rabbit pAb against HBc from Agilent Technologies is now out of production (23), and the pAbs from Abcam have a significant background in multiple biochemical assays. Although several mouse anti-HBc mAbs have been reported, the characteristics of their application in diverse biochemical assays are rarely evaluated (14, 15). Recently, a mouse mAb#7 binding to the C-terminal domain of HBc could be applied in the western blot, IFA, and immunoprecipitation assay. However, the immune spot assay is not evaluated and cytoplasmic HBc in HBV infection is rarely identified in IFA data (32). In this study, we identified a series of human anti-HBc mAbs, particularly cAbA1, cAbD4, and cAbF9 exhibited a broadly cross-genotypic recognition and could be applied in multiple biochemical assays. Furthermore, cAbA1, cAbD4, cAbF9, and the clinical agent MAB-0899 were evaluated to detect the HBc in chronic HBV liver tissues in immunohistochemistry (IHC) assay, head to head. As cAbA1, cAbD4, and cAbF9 are human antibodies, endogenous human antibodies may interfere with the recognition of secondary HRP-conjugated anti-human polyclonal antibodies. Thus, HRP-conjugated cAbA1, cAbD4, and cAbF9 were directly used to detect HBc in the IHC assay. cAbA1, cAbD4, and cAbF9 identified the HBV-infected hepatocytes in various degrees. Particularly, the signal-to-noise ratio of cAbA1 without process optimization was nearly comparable to that of the commercial assay MAB-0899 in this clinical test (Fig. S10). Thus, these three anti-HBc mAbs hold great progress for the application in HBV-related detection and might be widely applied in clinical tests after transforming the human constant region to a mouse or rabbit constant region in the future.

HBc dimer with hydrophobic interactions and an intermolecular disulfide C61-C61 can assemble T = 3 or T = 4 symmetric icosahedrons (4, 33). Several positions of HBc substituted by alanine or replacing residues 78–83 a.a. with other amino acids is generally not thought of as affecting the assembly of capsids (17, 34). However, a D78S substitution of HBc has been reported to cause dimer destabilization and structural deformation (29), which may not just affect the single D78 recognized by mAbs. In the binding epitope analysis, Group I mAbs mainly recognized the residues 20–22 a.a. region. However, the D78A positive proportion of detection by Group I mAbs was obviously lower than that detected by cAbA1, suggesting that the D78A mutation of HBc might affect both the D78 site and the other sites interacting with mAbs. It has been reported that P20A, D78A, and P130A mutations of HBc cannot support the HBV RNA reverse transcription and virion secretion (8). Therefore, we speculated that P20A and P130A mutations might also influence the recognition of mAbs by the conformation change of capsids. Indeed, the P20A mutation of HBc led to an obvious reduction of the recognition by Group I and Group III mAbs. Several studies have revealed that mAbs recognized residues 20–22 a.a. also bound to residues 127–131 a.a. of HBc (18, 28). However, P130A alongside R127A and A131R mutations had no effect on the recognition of 12 human anti-HBc mAbs identified in this study. Further studies on the precise binding epitopes might be revealed by the cryo-EM or crystal structure of HBc complexed with these mAbs in the future. Overall, we recommended that both residues 20–22 a.a. within the α1-α2 arm and ‘‘immunodominant loop’’ (~residues 78–83 a.a.) of HBc represented the important epitopes recognized by this panel of human mAbs.

Given the limited scope of binding epitope identification in this study, there may be other key sites of HBc influencing the recognition of these human mAbs. For example, although the same amino acids at positions 20–22 and 77–78 of HBc are present in all HBV genotypes, the HBc of genotypes F and H was influenced by the recognition of cAbA1 in some degree. Further study on the key binding sites of mAbs may be performed by the technology of structural biology. In this study, a cryo-EM structure of HBV capsid-cAbD4 Fabs revealed that E77, D78, and S81 were the key binding sites of cAbD4. However, E77A mutation of HBc has no effect on the recognition of cAbD4, suggesting that the other interaction sites might counteract the effect of E77A mutation. This phenomenon was also found in other virus-specific mAbs. For example, the F456A or Y489A mutation in binding epitopes of EH8 mAb has no effect on the recognition of EH8 to SARS-CoV-2 spike (35). In other words, mAbs can sustain several mutations of virus appearing at the binding epitopes, which endow mAbs with a broadly cross-genotypic reactivity. Therefore, cAbD4 mAb could serve as an excellent and dependable detection candidate for various genotypes of HBV.

Several studies have reported the cryo-EM structures of HBV capsids complexed with anti-HBc mAbs (20, 21, 28, 36). Although the binding epitopes and characteristics may be revealed at a resolution of ~10 Å, detailed information regarding the capsid-mAb interface is currently lacking. In this study, we determined the high-resolution structure for the interacting region between cAbD4 Fab and HBc dimer. Due to the symmetric identification on both sides of a dimer tip, the ASUs exhibit some heterogeneity, allowing for the application of four binding models: Class 1 (A3B4/C3D4), Class 2 (A4B3/C4D3), Class 3 (A4B3/C3D4), and Class 4 (A3B4/C4D3). Based on this kind of novel and accurate classification of dimer units, we further improved the local resolution around the interface between cAbD4 Fab and HBc dimer, which provided more detailed information of their interactions.

In summary, the present study reported a series of human anti-HBc mAbs possessed broadly cross-genotypic activity and could be effectively utilized in multiple biochemical assays for HBc detection. Most mAbs exhibited strong recognition of HBc overexpression or live HBV infection in ELISA, western blot, flow cytometry, IFA, and immune spot assay, especially Group II mAbs (cAbA1, cAbD4, and cAbF9). The most potent and broad-spectrum cAbD4 mAb and its high-resolution structure complexed with HBc dimer could greatly facilitate the development and progress of HBV-related studies in the future.

### Limitations of the study

There are some potential limitations in this study. First, the application of these 12 human anti-HBc mAbs was evaluated by a limited range of biochemical assays, such as ELISA, western blot, flow cytometry, IFA, and immune spot assay. Second, the accurate binding epitopes of these human mAbs, except for cAbD4, were not identified by structural biology. More efforts could help to address these limitations in the future.

## MATERIALS AND METHODS

### Study approval and biological samples

This study was approved by the Ethics Committee of Shenzhen Third People’s Hospital, China (approval number: 2021-030). The peripheral blood mononuclear cells from two chronic HBV patients (Donor A and Donor B; HBsAg positive, HBsAb negative, HBeAg negative, HBeAb positive, HBcAb positive) with HBV genotype B infection were used for the anti-HBc antibody isolation. One core needle liver biopsy (Donor 1) and two liver tissues (Donor 2 and Donor 3) from chronic HBV patients diagnosed with liver fibrosis or cirrhosis underwent surgery at the Shenzhen Third People’s Hospital and were chosen for immunohistochemistry assays. All biological samples from chronic HBV patients were stored in the Biobank of Shenzhen Third People’s Hospital. All participants had provided written informed consent for sample collection and subsequent analysis.

### Protein, plasmid, and antibody

HBc_143_ containing 1–143 amino acids was purchased from Shenzen Immuthy Biotechnology. HBc plasmids of genotype A (AF305422.1), B (AB602818.1), C (AF461357.1), D (V01460.1), E (PP790594.1), F (AB036910.1), G (KX264500.1), H (AY090454.1), I (AF241411.1), and J (AB486012.1) were synthesized by GenScript Biotech Corporation. Commercial mouse monoclonal antibody C1-5 (SC-23945) and 10E11 (SC-23947) and rabbit polyclonal antibody against HBc (pAb, AB115992) were purchased from Santa Cruz Biotechnology and Abcam, respectively. Paired heavy-chain and light-chain plasmids of mAbs (cAbA1, cAbB4, cAbB8, cAbD4, cAbD8, cAbE2, cAbE5, cAbE11, cAbF5, cAbF9, cAbF12, cAbH8, and VRC01) were co-transfected into 293F cells, respectively. After 6 days, mAbs were purified from cell supernatants using protein A columns, and then quantified by NanoDrop and stored at −80°C before use. cAbA1, cAbB4, cAbB8, cAbD4, cAbD8, cAbE2, and cAbE11 were isolated from Donor A. cAbE5, cAbF5, cAbF9, cAbF12, and cAbH8 were isolated from Donor B. VRC01 was a HIV-1-specific mAb and used here as the negative control.

### Cell culture and virus

Hep AD38 human hepatoma cells harboring HBV genome (MF967563.1) were cultured in Dulbecco’s modiﬁed Eagle’s medium (DMEM, Gibco), supplemented with 10% heat-inactivated fetal bovine serum (FBS, Gibco) and 100 U/mL penicillin/streptomycin (Gibco) at 37°C in 5% CO_2_. HepG2-NTCP cells were maintained in DMEM supplemented with 10% FBS, 1% Hepes, 1% penicillin/streptomycin, and 1 µg/mL puromycin (ThermoScientiﬁc,) at 37°C in 5% CO_2_.

HBV particles generated from Hep AD38 were puriﬁed by sucrose density centrifugation. The Hep AD38 supernatants were loaded onto an ultracentrifuge tube containing 15% sucrose and then centrifuged in a SW32 rotor (Beckman) at 130,000 × *g* for 16 hours at 4°C. After centrifugation, the pellets containing the virus were resuspended in DMEM.

For HBV infection, HepG2-NTCP cells were seeded into collagen-I-coated 96-well plates and maintained in DMEM medium for 12–18 hours. Then these cells were infected with HBV in DMEM with 10% FBS, 4% PEG8000, and 2.5% DMSO at 37°C for 24 hours, and then washed with phosphate-buffered saline (PBS) and maintained in DMEM with 10% FBS, 1% penicillin/streptomycin, and 2.5% DMSO. The supernatant was collected and used for HBsAg and HBeAg detection every 2–3 days.

### Enzyme-linked immunosorbent assay

The 96-well plates were coated with HBc_143_ of genotype C (2 µg/mL) at 4°C overnight and then blocked with 2% bovine albumin and 5% skim milk in PBS for 1 hour at room temperature (RT). Fivefold diluted mAbs or rabbit pAb against HBc were added into wells. After 1 hour at 37°C, the plates were washed with PBST and incubated with 5,000-fold diluted HRP-conjugated second antibody, including Goat anti-Human, Goat anti-rabbit IgG, or Goat anti-mouse IgG (ZSGB-BIO) secondary antibody at 37°C for another 1 hour. The enzymatic reaction was then developed with TMB substrate (Sangon Biotech) at RT for 20 minutes and terminated by the addition of 2 M H_2_SO_4_. The optical density was detected at 450 nm. The half maximal effective concentration (EC_50_) values were calculated using GraphPad Prism 8 software by log (agonist) vs response -- Variable slope (four parameters) model.

For competition ELISA, the coating and blocking of plates were the same as the above protocol. Then, PBS or 10 µg/mL mAbs mixed with HRP (Abcam) conjugated cAbA1, cAbF5, cAbB4, or cAbH8 in equal volumes were added into the plates and incubated at 37°C for 1 hour. The detection was also the same as the above protocol.

### Western blot assay (WB)

HBc-overexpressed 239T cells or HBV-infected HepG2-NTCP cells were treated with loading buffer containing 0.5% SDS and 40 mM DTT at 100°C for 10 min. After that, these samples were loaded into 12.5% sodium dodecyl sulfate polyacrylamide gel electrophoresis (SDS-PAGE) and then transferred to polyvinylidene difluoride (PVDF) membrane using the Mini-PROTEAN Tetra System (Bio-Rad). The membranes were blocked with 5% skim milk for 1 hour at RT, followed by the addition of mAbs or rabbit pAb overnight at 4°C and incubated with HRP-conjugated Goat anti-Human, Goat anti-rabbit IgG, or Goat anti-mouse IgG (ZSGB-BIO) for 1 hour at RT. The protein bands were visualized with Clarity Western ECL Substrate and a ChemiDoc MP Imaging System (Bio-Rad).

### Immunofluorescence assay (IFA)

293T cells transfected with HBc plasmid or HepG2-NTCP cells infected by HBV were fixed with 4% paraformaldehyde for 30 minutes at RT, permeabilized with 0.2% Triton X-100 in PBS for 20 minutes at RT. After being blocked with QuickBlock Blocking Buffer (Beyotime) for 1 hour at RT, the cells were added with 10 µg/mL mAbs or pAb at 4°C overnight and then incubated with Alexa Fluor 488-conjugated secondary antibody (Thermo Fisher Scientific) for 1 hour at RT. Finally, the cells were stained with 2-(4-amidinophenyl) indole-6-carboxamidine dihydrochloride (DAPI, Beyotime) for 15 minutes at RT. Microscopic images were captured under a Leica DMi8 inverted microscope (Leica Microsystems).

### Flow cytometry

293T cells expressed HBc proteins of genotypes A–J or HepG2-NTCP cells infected with HBV were fixed and permeabilized using Fixation/Permeabilization Solution Kit (BD Biosciences) according to the manufacturer’s instruction, and then stained with 5 µg/mL mAbs or pAb for 30 minutes at 4°C, followed by the incubation of Alexa Fluor 647-conjugated secondary antibody (Thermo Fisher Scientific) for another 30 minutes at 4°C. Dead cells were excluded by the LIVE/DEAD Fixable Violet Dead Cell Stain kit (Thermo Scientific). Data were acquired by FACSymphony A3 (BD Biosciences) and analyzed by FlowJo software V10.9 (BD Biosciences).

### Immune spot assay

HepG2-NTCP cells were infected with 1,800, 600, and 200 genome equivalent copies of HBV per cell, respectively, in the presence of 4% PEG8000 and 2.5% DMSO, and then cultured in DMEM medium in the presence of 10% FBS and 2.5% DMSO. After 7 days, the infected cells were fixed with 4% paraformaldehyde, permeabilized with 0.1% Triton X-100 in PBS, and then blocked with QuickBlock Blocking Buffer (Beyotime) for 1 hour at RT. Then cells were stained with 10 µg/mL mAbs or rabbit pAb overnight at 4°C, followed by the incubation of HRP-conjugated Goat anti-Human, Goat anti-rabbit IgG, or Goat anti-mouse IgG (ZSGB-BIO) secondary antibody for 1 hour at RT. Finally, the reactions were developed with KPL TrueBlue Peroxidase substrates (Seracare Life Sciences), and the HBV-infected cells were captured by using Cytation 7 Cell Imaging Multimode Reader (BioTek).

### Immunohistochemistry assays

Formalin-ﬁxed parafﬁn-embedded liver sections were detected by a clinical assay MAB-0899 (clone MX104) according to the manufacturer’s instructions which was purchased from Maixin Biotechnology Development (Fuzhou, China). Others were stained by 7 µg/mL of HRP-conjugated cAbA1 (cAbA1-HRP), 20 µg/mL of HRP-conjugated cAbD4 (cAbD4-HRP), 10 µg/mL of HRP-conjugated cAbF9 (cAbF9-HRP), and 10 µg/mL of C1-5 antibody. Then, the C1-5-stained liver sections were followed by secondary HRP-conjugated goat anti-mouse IgG. All the sections were developed with DAB assays and visualized by Motic VM1000 (Motic Electric Group).

### Binding epitope analysis

Alanine mutation in HBc of genotype D residues: P20A, D22A, P25A, D29A, T70A, V74A, E77A, D78A, P79A, D83A, L84A, R127A, P130A, and A131R, were conducted, respectively, using Mut Express MultiS Fast Mutagenesis Kit V2 (Vazyme). These HBc mutations were expressed in 293T cells and detected with mAbs or pAb by Flow cytometry assay after 48 h.

Bril fused different HBc truncations with flag tags were recombined using the ClonExpress MultiS One Step Cloning Kit (Vazyme) and then transfected into 239T cells. After 48 hours, these cells were collected and detected using human mAbs cAbA1, cAbB4, cAbD4, and cAbF9 in western blot assay.

### Cryo-EM sample preparation and data collection

For HBc-cAbD4 cryo-grid preparation, HBc was mixed with Fab of cAbD4 antibody at 4°C, at final concentrations of 3 and 0.5 mg/mL, respectively. After incubation on ice, 3 µL of the HBc-cAbD4 complex was applied to freshly glow-discharged holy carbon grids (Quantifoil Cu R2/1) in Vitrobot Mark IV at 4°C and 100% humidity. Grids were blotted for about 3.5 s after waiting for 2 s. Data were collected using a Titan Krios microscope with a Falcon IV. Fractions were automatically collected using EPU software, with a pixel size of 0.82 Å/pixel. Exposures were performed with a total dose of 60 e^-^/Å^2^. Defocus ranges from −1.5 to −2.5 µm.

### Cryo-EM data processing and model building

A total of 4,173 fractions were aligned and averaged using MotionCor3. The contrast transfer function parameters were determined using CTFFIND4. The thresholds for CTF fit resolution and astigmatism of micrographs were set at 5.5 and 1,000 Å, respectively, to screen out the good micrographs. Coordinates for particles were auto-picked out using the Blob picker job in cryoSPARC, resulting in a total of 817,068 particles. Bin*4 images for the particles were extracted to perform the subsequent calculation. After several rounds of 2D classification, 554,907 good particles were selected. Homo Refine was executed with the I symmetry using the EMD_14866 as the initial volume. Particles were imported into RELION3.1 to execute 3D classification. A total of 115,656 particles were selected and extracted with binning 2. After that, Class3D was performed. A total of 16,919 particles were selected and un-binned images were extracted to perform the Refine3D job with I symmetry, resulting in a density map at 3.44 Å. To improve the qualities of the density map at the region of cAbD4 Fab, the asymmetric unit (ASU) was masked out. Particles were expanded with I symmetry and subtracted with the ASU mask to keep the ASU signals in RELION3.1. The resulting particles were used to perform 3D classification. After that, good particles were imported to cryoSPARC and performed local refinement step-by-step. Four density maps were obtained at the resolution of 3.68, 3.22, 3.61, and 3.51 Å, respectively. Local resolutions were calculated in cryoSPARC. Post-processing of maps was performed by deepEMhancer software.

The structure for HBV core (PDB: 3J2V) or cAbD4 Fab predicted by AlphaFold2 was used as the initial model to fit in the density maps using UCSF chimera. The amino acids were then manually adjusted in Coot. The results were further refined by real-space refinement program in Phenix. After several rounds of the refinement cycles, the quality of the final atomic model was evaluated using MolProbity. Details of the data collection, processing, and model building are presented in Fig. S5 and Table S1.

### Amino acid sequences of cAbD4

#### Heavy chain

QVQLVESGGGVVQPGRSLRLSCAASGFNFNKFGMHWVRQVPGKGLEWLTYIWYDGSNADYVDSVKGRFTISRDNSINTLYLQMNSLRADDTAVYFCARGFYDSSSLESWGQGALVIVSS

#### Light chain

DIVMTQSPLSLAVTPGEPASISCRSSQTLLHNNGYNYFSWYLQKPGQAPQLLIYLGSNRAPGVSDRFSGSGSGTSFTLEISRVEAEDVGVYYCMQGRHTPWTFGQGTKVEIK

### Statistical analysis

Statistical analysis was performed with GraphPad Prism 8.0 (GraphPad Software). Differences among multiple groups were evaluated by one-way analysis of variance (ANOVA). Data were presented as the mean ± standard deviations (SD). *P* < 0.05 was considered statistical significance and denoted with asterisks (*P < 0.05, **P < 0.01, ***P < 0.001, ****P < 0.0001).

## Data Availability

The structure coordinates for the HBV capsid-cAbD4 Fab complex, the asymmetric unit of the HBV capsid-cAbD4 Fab complex, and the HBc dimer-AB and Fab-hl complexes have been deposited in the Protein Data Bank under accession codes 8ZRE, 8ZRH, and 8ZRR, respectively. The corresponding cryo-EM density maps have been deposited in the Electron Microscopy Data Bank under accession numbers EMD-60395, EMD-60396, and EMD-60403. This paper does not report the original code. Any additional information required to reanalyze the data reported in this paper is available from the lead contact upon request.

## SUPPLEMENTAL MATERIAL

Supplemental material jvi.01694-24-s0001.pdf Figures S1 to S10; Table S1.

ASM does not own the copyrights to Supplemental Material that may be linked to, or accessed through, an article. The authors have granted ASM a non-exclusive, world-wide license to publish the Supplemental Material files. Please contact the corresponding author directly for reuse.
