# Supplementary material for: The characterization and structural basis of a human broadly binding antibody to HBV core protein
Source: J Virol. 2024 Nov 27;99(1):e01694-24. doi: 10.1128/jvi.01694-24 (PMC11784010; doi:10.1128/jvi.01694-24)
Supplement: Supplemental material — Figures S1 to S10; Table S1. [file jvi.01694-24-s0001.pdf]

# Supplementary information

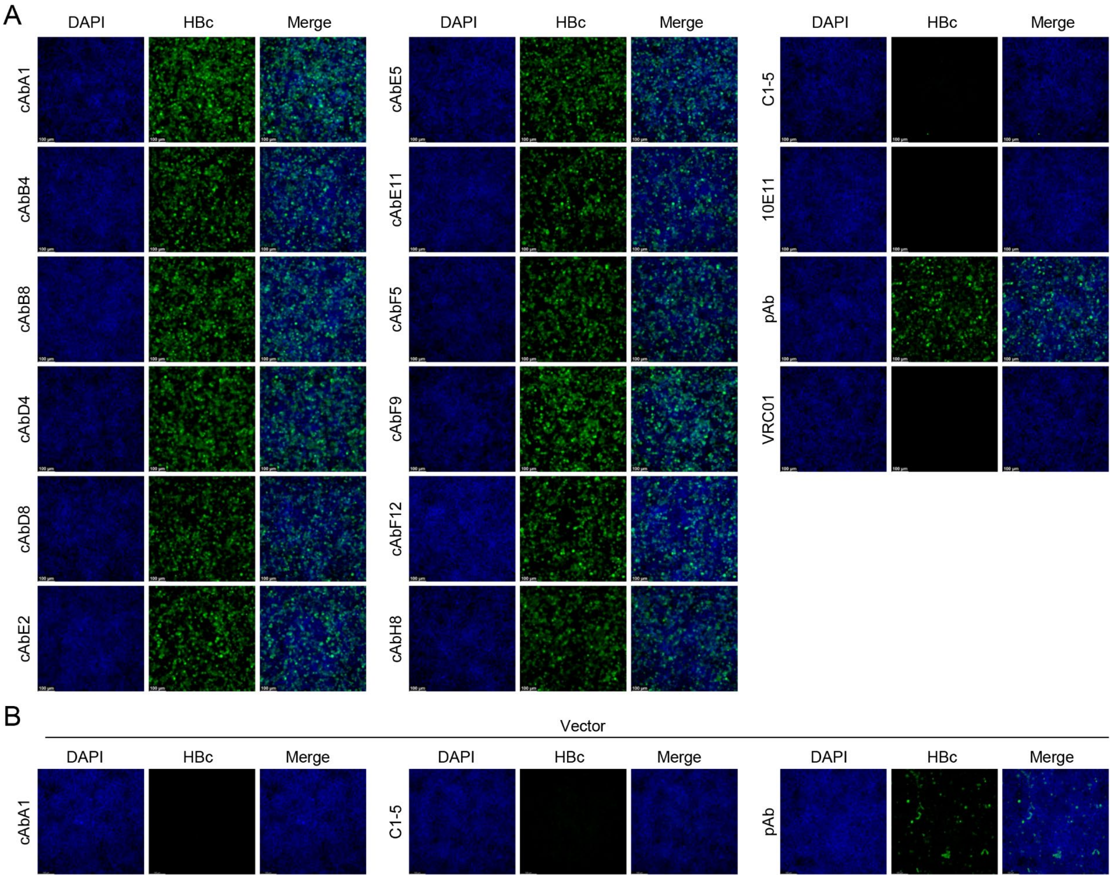


**Figure S1. Immunofluorescence analysis of human anti-HBc mAbs in the detection of HBc overexpression.**

Full-length HBc protein of HBV genotype C (**A**) and vector control (**B**) were transfected into 293T cells respectively. After 48 hours, cells were fixed, penetrated and then incubated with 5 μg/mL human anti-HBc mAbs, C1-5, 10E11, and pAb respectively. At last, the cells were detected by Alexa fluor 488 conjugated second antibody. Scale bar: 100 μm. The experiment was independently performed at least twice and one representative result was shown.

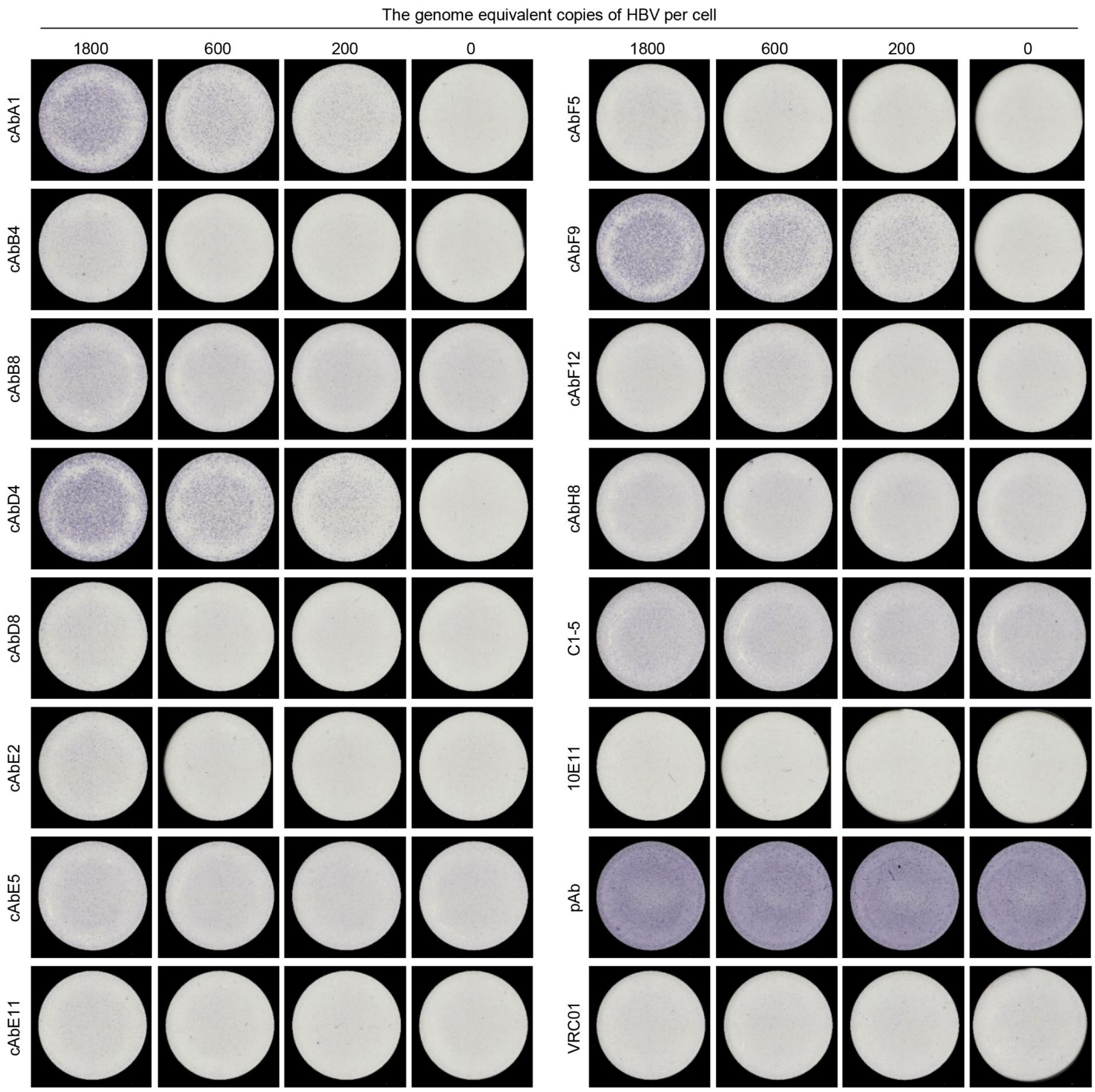


**Figure S2. The immune spot assay analysis of human anti-HBc mAbs for the detection of HBV infection.**

HepG2-NTCP cells were infected with 1800, 600 and 200 genome equivalent copies of HBV per cell respectively. After 5-7 days, the single HBV-infected cell was identified by 12 human anti-HBc mAbs, C1-5, 10E11, and pAb respectively with HRP-conjugated second antibodies. The experiment was independently performed at least twice and one representative result was shown.

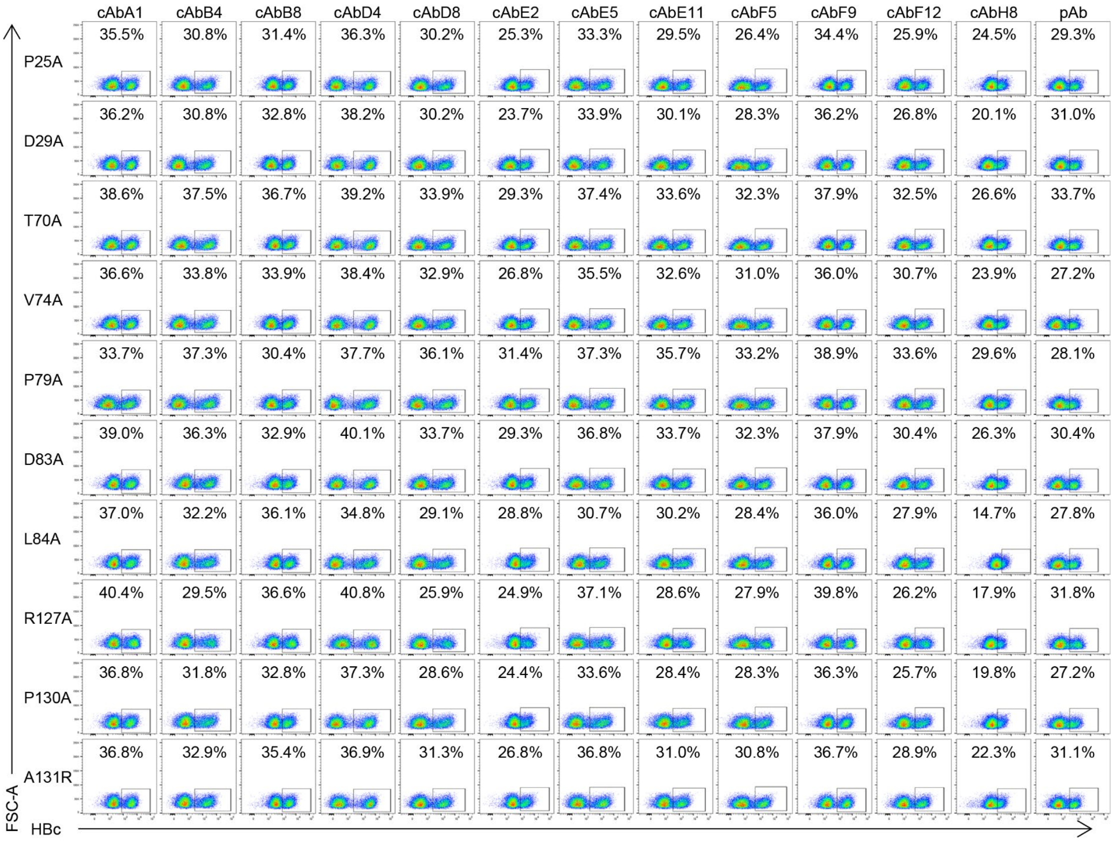


**Figure S3. The key binding sites of human anti-HBc mAbs detected by alanine scanning mutagenesis.**

The residues 25, 29, 70, 74, 79, 83, 84, 127, 130 of HBc from genotype D mutated to alanine and 131 mutated to arginine were overexpressed in 293T cells and detected by human anti-HBc mAbs in flow cytometry analysis. The experiment was independently performed at least twice and one representative result was shown.

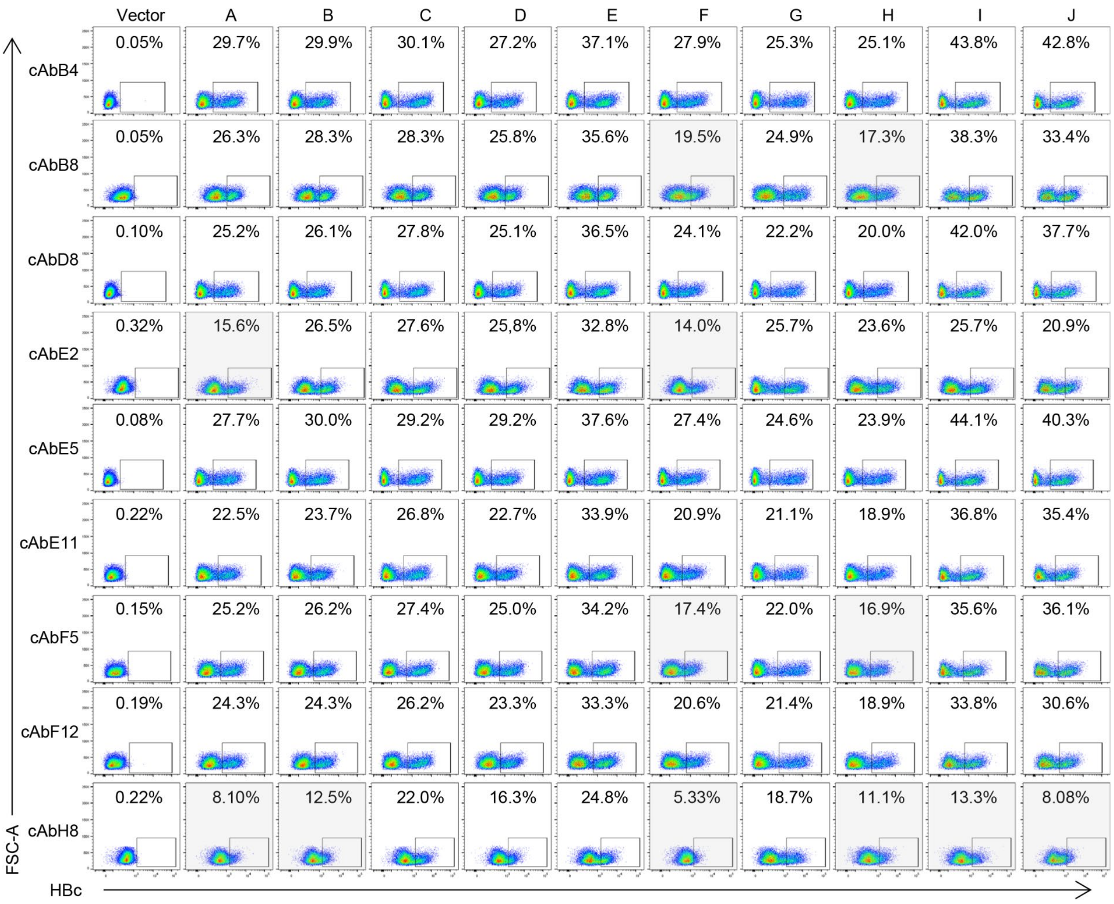


**Figure S4. The HBc detection of genotypes A-J by human anti-HBc mAbs.**

The reactivity of human mAbs cAbB4, cAbB8, cAbD8, cAbE2, cAbE5, cAbE11, cAbF5, cAbF12 and cAbH8 to detect HBc of genotypes A-J were evaluated by flow cytometry. The experiment was independently performed at least twice and one representative result was shown.

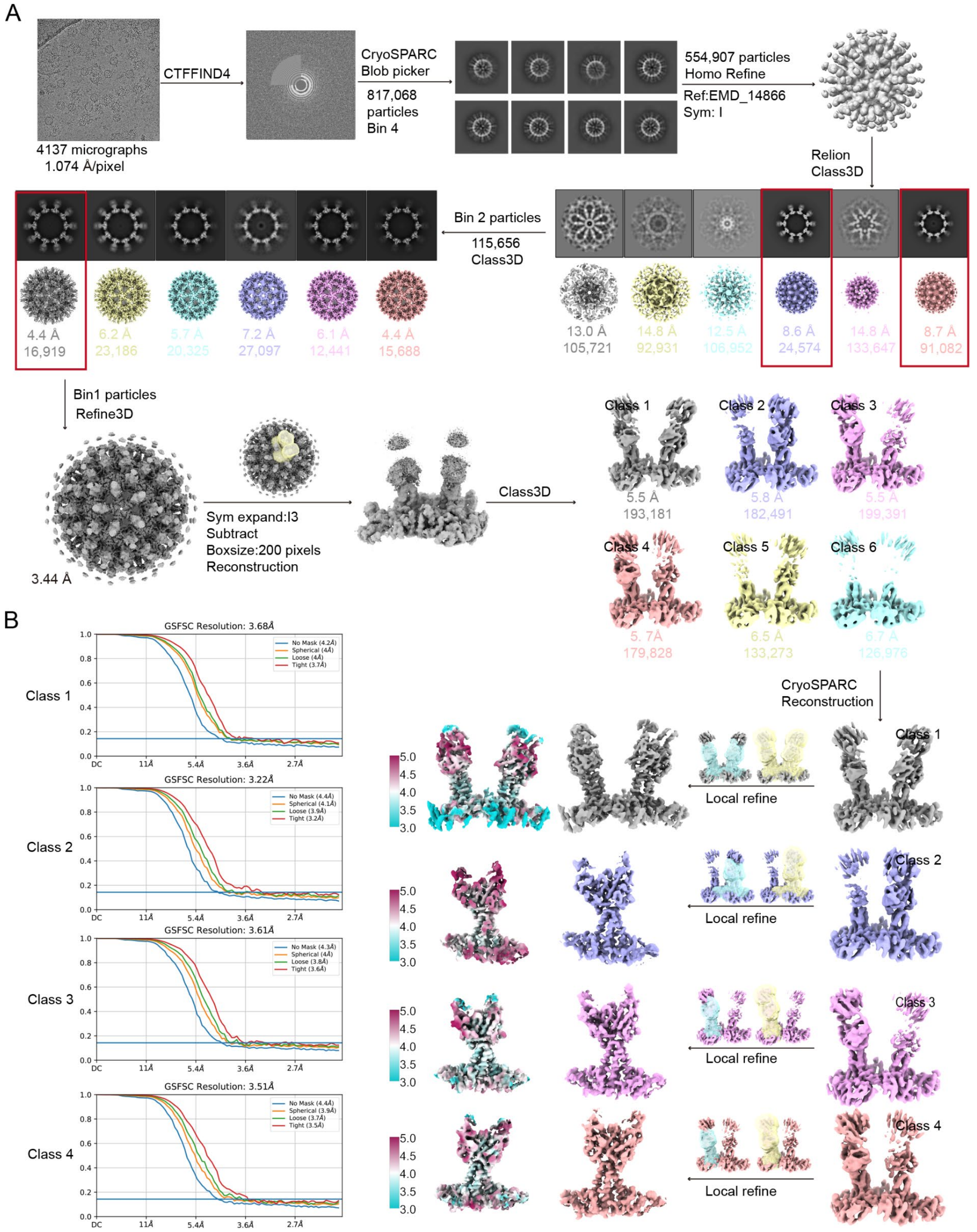


**Figure S5. Cryo-EM image-processing workflow for HBV capsid-cAbD4 Fabs complex.**

(**A**) Workflow for single-particle analysis of the HBV capsid-cAbD4 Fabs complex, including representative cryo-EM micrograph, CTF estimation results, 2D and 3D classification results, particle subtraction, local refinements, and local resolution analysis. (**B**) FSC curves for different density maps.

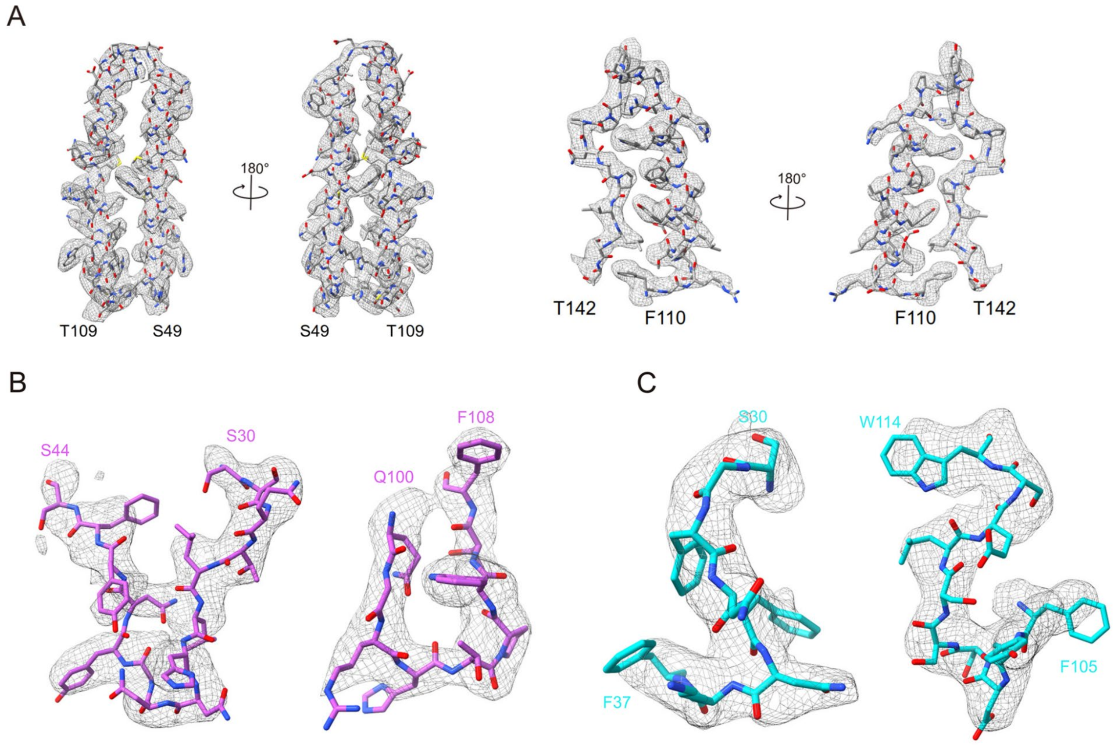


**Figure S6. Cryo-EM density maps of the HBV capsid-cAbD4 Fabs complex.**

(**A**) Density maps for residues S49-T109 and F110-T142 of the HBc molecule. (**B**) Density map of the light chain of cAbD4 Fab, encompassing residues S30-S44 and Q100-F108. (**C**) Density map of the heavy chain of cAbD4 Fab, showing residues S30-F37 and F105-W114.

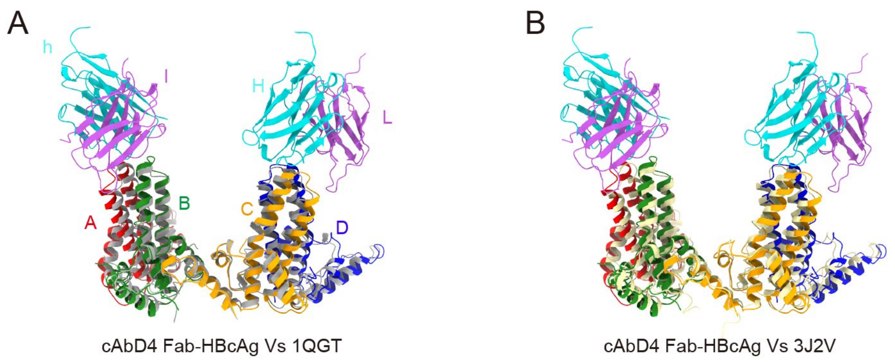


**Figure S7. The asymmetric units (ASUs) structure of HBV capsid.**

(**A, B**) Structure comparison between the ASUs of the cAbD4 Fab-HBc complex and the apo HBc structures determined by crystal diffraction (PDB: 1QGT) or cryo-EM (PDB: 3J2V). The cAbD4 Fab-HBc complex determined in this study is displayed in color, while the structures for 1QGT and 3J2V are colored in grey and yellow, respectively.

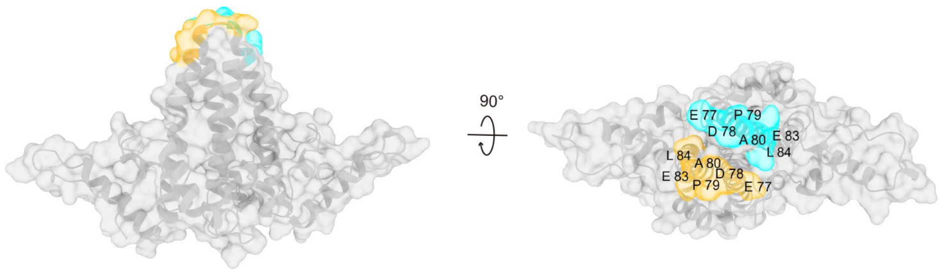


**Figure S8. Footprint of 3105 Fabs on the HBc dimer tip.**

The footprint of chain A is represented by orange, and the footprint of chain B is represented by blue. The relevant amino acids are marked accordingly.

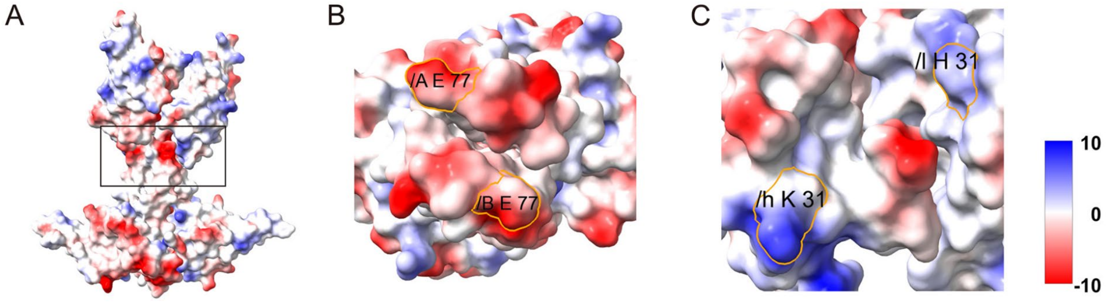


**Figure S9. The residues E77 formed salt bridges with the residues K31 and H31 of mAbs.**

(**A**) Coulombic electrostatic potential of the dimer-AB and Fab-hl complex. (**B**) Top view of the dimer-AB complex. (**C**) Bottom view of the Fab-hl complex. The electrostatic potential is calculated using ChimeraX. In the electrostatic potential maps, the two negatively charged amino acids (E77 in both chain A and B) and the two positively charged amino acids (K31 in the heavy chain and H31 in the light chain) are highlighted and circled.

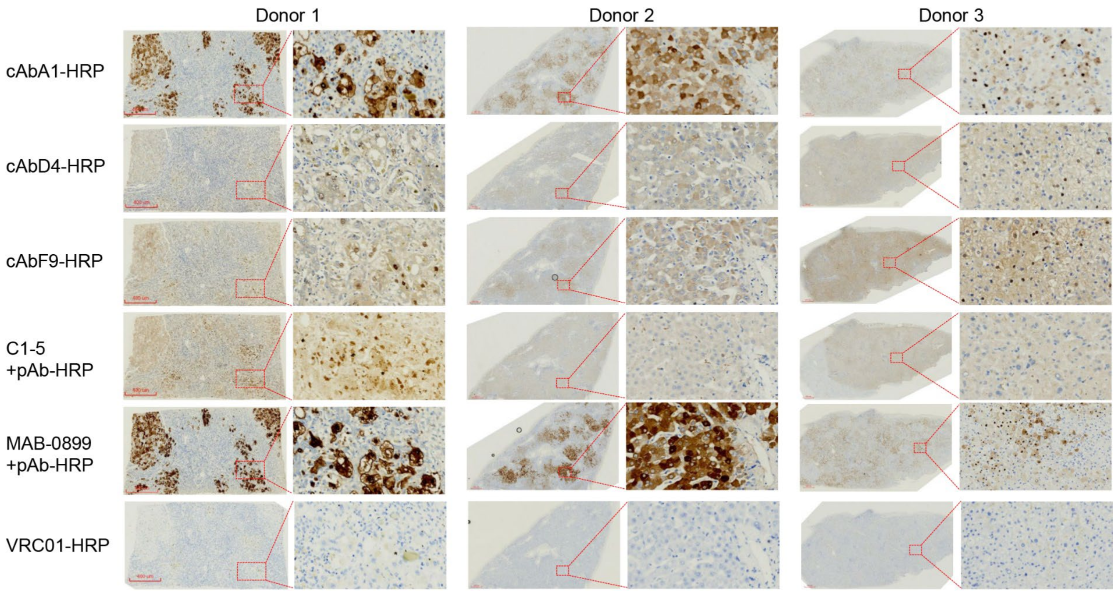


**Figure S10. Intrahepatic viral antigens were detected by identified anti-HBc mAbs in chronic HBV liver tissues.**

Formalin fixed paraffin embedded human liver sections were detected by MAB-0899, cAbA1-HRP, cAbD4-HRP, cAbF9-HRP or C1-5 respectively in immunohistochemistry assays. Scale bar: 400 μm.

**Table S1. Cryo-EM data collection and processing, model building and refinement statistics.**

| Iterms \ Data set | HBcAg-cAbD4 Fab complex | | |
|---|---|---|---|
| Microscope | FEI Titan Krios | | |
| Camera | Falcon IV | | |
| Voltage(kV) | 300 | | |
| Automation software | EPU | | |
| Total dose (e–/Å2) | 60 | | |
| Frames | 40 | | |
| Defocus range (μm) | -1.5-2.5 | | |
| Pixel size (Å/pixel) | 1.074 | | |
| Micrographs used (no.) | 4,173 | | |
| Initial particles images | 817,068 | | |
| Density map | Dimer-AB and Fab-hl | ASU of HBcAg-cAbD4 Fab | HBcAg-cAbD4 Fab |
| Symmetry imposed | C1 | C1 | I3 |
| Final particles images | 199,391 | 193,181 | 16,919 |
| Resolution(Å) | 3.61 | 3.68 | 3.44 |
| EMDB | EMD-60403 | EMD-60396 | EMD-60395 |
| Nonhydrogen atoms | 4065 | 8130 | 8085 |
| Protein residues | 516 | 1032 | 1026 |
| Ligands | 0 | 0 | 0 |
| Length (Å) (# > 4σ) | 0.009(17) | 0.009(34) | 0.009(54) |
| Angles (°) (# > 4σ) | 1.221(5) | 1.221(10) | 0.953(40) |
| MolProbity score | 2.22 | 2.34 | 2.23 |
| CαBLAM outliers (%) | 1.8 | 1.8 | 3.03 |
| Clashscore | 8.98 | 13.28 | 9.91 |
| Rotamer outliers (%) | 3.81 | 3.48 | 3.16 |
| C-beta outliers (%) | 0.0 | 0.0 | 0.0 |
| Favored (%) | 95.67 | 95.67 | 95.14 |
| Allowed(%) | 3.54 | 3.54 | 4.37 |
| Outliers(%) | 0.79 | 0.79 | 0.5 |
| PDB | 8ZRR | 8ZRH | 8ZRE |
